# Fungal necromass drives MAOC accrual in SOC pools across altitudinal gradients of *Potentilla parvifolia* in the Qilian Mountains, Northwest China

**DOI:** 10.1128/spectrum.01605-25

**Published:** 2025-10-27

**Authors:** Haining Gao, Shengsong Wu, Xiaoli Wang, Xiaoyi Liu, Lijie Liao, Yong Zhang, Yong Chen, Qi Feng

**Affiliations:** 1 College of Life Science and Engineering, Hexi University 74786, Zhangye, China; 2 School of Life Sciences, Lanzhou University 12426, Lanzhou, China; 3 School of Biological and Pharmaceutical Engineering, Lanzhou Jiaotong University 12383, Lanzhou, China; 4 Key Laboratory of Ecohydrology of Inland River Basin, Northwest Institute of Eco-Environment and Resources, Chinese Academy of Sciences, Lanzhou, China; Instituto de Ecología, A.C. (INECOL), Pátzcuaro, Michoacán, Mexico

**Keywords:** Qilian Mountains, *Potentilla parvifolia*, microbial necromass carbon, soil organic carbon fractions, phospholipid fatty acid, soil microbial community

## Abstract

Soil organic carbon (SOC) plays a critical role in global carbon cycling and climate regulation, particularly in high-altitude permafrost regions. However, the impact of altitudinal gradients of alpine shrubs on SOC fractions remains poorly understood. In this study, we evaluated the rhizosphere SOC fractions and microbial biomass of *Potentilla parvifolia* along an altitudinal gradient (3,204, 3,350, 3,550, and 3,650 m). Our findings revealed that *P. parvifolia* significantly increased gram-positive bacterial and fungal biomass at medium and low altitudes (3,204, 3,350, and 3,550 m), enhancing the contribution of mineral-associated organic carbon (MAOC) to total SOC compared to bare soil. Moreover, SOC accumulation was primarily driven by the buildup of microbial necromass carbon, particularly fungal necromass carbon, within the MAOC fraction. These results improve our understanding of how altitudinal gradients influence SOC dynamics and microbial mechanisms, providing a scientific basis for developing effective bioprotection strategies to conserve high-altitude ecosystems under global climate change.

**IMPORTANCE:**

This study addresses critical knowledge gaps in understanding how altitudinal variation of shrubs affects soil carbon dynamics in the Qilian Mountains' seasonal permafrost. Investigating the redistribution between particulate organic carbon and mineral-associated organic carbon, along with microbial necromass (fungal vs bacterial), is vital for predicting alpine carbon-climate feedbacks. Shrub encroachment into higher elevations may alter vegetation-derived carbon inputs and decomposition pathways, potentially destabilizing historically protected permafrost carbon stocks. The unique freeze-thaw cycles in seasonal permafrost likely modulate microbial processing of necromass into stable carbon pools, a mechanism poorly understood in cold biomes. By elucidating altitude-dependent shifts in carbon fractions and microbial legacy effects, this research provides mechanistic insights into vegetation-mediated carbon sequestration under climate change. Findings will inform models predicting permafrost carbon vulnerability and guide alpine ecosystem management strategies in this climate-sensitive headwater region critical for downstream water security.

## INTRODUCTION

Soil constitutes the second most significant carbon reservoir globally, with carbon present mainly as a form of organic carbon (1, 2). Because of its large scale and potentially long residence time, the production and degradation of soil organic carbon (SOC) can significantly affect the sequestration and release of carbon dioxide, thus directly affecting short-term climate regulation (3, 4). SOC is primarily composed of particulate organic carbon (POC) and mineral-associated organic carbon (MAOC), which have different characteristics and functions (5). The destiny of SOC beneath vegetative canopies is predominantly governed by MAOC (6), which is largely protected from decomposition through encapsulation or the shielding effect within mineral-associated soil particles (5). POC consists of labile, yet relatively recalcitrant, light fractions (5). It is crucial to investigate how cover shrubs impact POC and MAOC to comprehend the processes of organic carbon sequestration. Furthermore, plant detritus is believed to be the main source of SOC; however, recent findings suggest that organic carbon is predominantly supplied by microorganisms (7–9), where SOC owes 50%–80% of its composition to microbial necrotic mass (10). Over time, the remains of deceased microbial cells build up in the soil, creating one of Earth’s most substantial organic matter reservoirs.

As the most varied assembly within the biosphere, the soil microbiome represents no less than 25% of the planet’s biodiversity (11). A multitude of species, including bacteria, fungi, and microeukaryotes, thrive beneath the surface; however, only a fraction, in the hundreds of thousands, has been meticulously cataloged (12). The prevalence of bacteria and fungi significantly surpasses that of other microbial species; these groups overwhelmingly constitute the biomass and diversity of the soil microbial community (13). Moreover, these organisms play a pivotal role in the SOC cycle, and their necromass contributes to the stabilization of soil organic matter. They possess multiple enzymes involved in carbon degradation, such as glycoside hydrolases and glycosyltransferases, which hydrolyze sugars and glycoconjugates and facilitate the synthesis of glycosidic bonds to yield glycosides (14, 15). Soil bacteria exhibit robust decomposition capabilities, engaging in the breakdown of deceased fauna, flora, and fungal hyphae.

Bacteria classified as gram positive and gram negative demonstrate distinct selectivity for carbon utilization (16, 17). K-selected microbes (normally gram-positive bacteria) have shown high resource utilization and low growth rates, whereas r-selected microbes (normally gram-negative bacteria) have shown low energy use but rapid growth and reproduction (18). K-selected microbes usually grow slowly, have high enzyme-substrate affinity, and use recalcitrant carbon; therefore, they generally have higher stress resistance, whereas r-selected microbes usually use labile carbon (19, 20). Fungi are frequently implicated in the gradual breakdown of resistant organic materials and possess enzymes capable of metabolizing intricate carbon-dense substrates (21, 22). Moreover, the architecture of fungi significantly enhances SOC sequestration because their mycelial networks, characterized by expansive surface areas and narrow diameters, penetrate regions beyond the reach of plant roots, including tiny soil pores and cores of soil clumps (23). The vast majority of terrestrial plants engage in symbiotic associations with mycorrhizal fungi, which significantly influence soil structure and, by extension, carbon dynamics within the soil (24).

Alpine ecosystems, particularly those in arid northwestern China like the Qilian Mountains, are highly sensitive to global climate change. Nestled in the arid northwest of China, the Qilian Mountains stand at the crossroads where the Qinghai–Tibet, Inner Mongolia–Xinjiang, and Loess Plateaus converge (25). It serves as a critical area for preserving biodiversity in China, acts as an essential access point for the International Alpine Germplasm Resource Bank, and provides a passage for wildlife (26). Global warming is a serious concern. It affects soil freezing and thawing processes, which are important for biogeochemical cycles in terrestrial ecosystems (27). It also alters the physical, chemical, and biological properties of the soil, including water content, pH, and conductivity, as this repetitive process ultimately alters nutrient and carbon cycling (28, 29). *Potentilla parvifolia* (Rosaceae) is a dominant shrub species widely distributed in alpine and subalpine zones of the Qilian Mountains (10). Recent studies have demonstrated that this species strongly influences soil microbial communities and their environmental interactions along altitudinal gradients (10). Shrub expansion or dominance shifts are increasingly recognized as critical drivers of soil microbial diversity and function in alpine ecosystems (6, 11). These vegetation–microbe feedbacks may have cascading effects on nutrient cycling and ecosystem multifunctionality under ongoing climate change (11). The migration of *P. parvifolia* is expected to affect soil microorganisms, thereby influencing biogeochemical cycles. However, the effects of *P. parvifolia* migration on the SOC cycle in the study area remain insufficiently explored.

Soil microorganisms are fundamental drivers of soil organic carbon dynamics and constitute a substantial, yet incompletely cataloged, portion of terrestrial biodiversity. While broad reviews emphasize their central ecological roles, recent studies show that vegetation structure (e.g., shrub presence or expansion) and elevation interact to reshape microbial composition and functional potential in alpine systems. For example, shrub expansion has been linked to increases in oligotrophic bacteria and ericoid mycorrhizal fungi in alpine grasslands, along with reductions in soil nitrogen availability and soil respiration (1). In subtropical alpine grassland, shrub encroachment enhances the diversity, stability, and complexity of arbuscular mycorrhizal fungi (AMF) networks compared to natural grasslands (2). In Qinghai–Tibetan Plateau grasslands, shrub encroachment alters both bacterial community composition and predicted functions, including in deeper soil layers (3). In addition, expansion of woody shrubs across alpine elevation gradients has been shown to shift soil fungal diversity and community distribution (4). However, direct evidence for an altitudinal range shift of *P. parvifolia* is lacking; therefore, our study frames the objectives in terms of elevation-dependent differences in rhizosphere vs bulk soil microbial communities (abiotic filtering plus host selection), a perspective consistent with recent alpine shrub–microbe interaction studies.

## MATERIALS AND METHODS

### Study area

The research location was situated within the Binggou Basin of Qilian County, Qinghai Province, which falls within the coordinates of 100°11′–100°23′E; 38°02′–38°10′N. Recognized as a pivotal region for ecological conservation in China’s economic blueprint (30), this area marks the intersection of the Tibetan, Mongolian, and Loess Plateaus. Characterized by the intersection of monsoonal and westerly wind currents, it exhibits the climatic traits of a continental and plateau environment. The mountainous region experiences significant variations in altitude and thermal conditions, leading to a pronounced zonal vegetation distribution that aligns with the elevational gradient descending from the coast (30). Spanning an elevation from 2,600 to 4,401 m above sea level, the Qilian Mountains exhibit a frigid average annual temperature of −0.1°C, coupled with an average annual precipitation of 412.6 mm (31). The study region, which has a continental alpine and semi-humid climate, predominantly features alpine meadow and subalpine shrub meadow soils.

### Experimental design and soil sampling

Soil specimens were gathered from different elevation points (3,204, 3,550, 3,650, and 3,750 m) within the central region of the Qilian Mountains. For each elevation, the rhizosphere soil surrounding *P. parvifolia* served as the experimental subject, while the barren soil was used as a baseline. Five discrete sampling sites were established at each altitude. Employing a plum bloom sampling technique, the soil was extracted from a depth of 5–15 cm, with the above-ground vegetation and the 5 cm litter layer initially excised. The soil was thoroughly mixed and transferred to a sterile aluminum container. The root system was removed and shaken gently. Detached soil blocks (soil 1 cm from the root system) and rhizosphere soil were collected using sterile gloves. Five soil samples were gathered in triplicate for consistency. Each sample was ground to evaluate its physicochemical characteristics.

### Soil organic carbon and physicochemical analyses

SOC was determined by the potassium dichromate external heating-oxidation method. Soil total nitrogen (TN) was measured using the semi-micro Kjeldahl method, while soil total carbon (TC) was determined via dry combustion at high temperature using an elemental analyzer (32). Microbial biomass carbon (MBC) was quantified using the chloroform fumigation-extraction method (24 h fumigation, 0.5 M K_2_SO_4_ extraction) (33).

### Phospholipid fatty acid analysis

Four samples were analyzed at each depth and altitude. For the phospholipid fatty acid (PLFA) analysis, we collected duplicate samples and stored them at −20°C until further extraction. After freeze-drying the soil samples, the Bligh and Dyer method (1959) is utilized to extract PLFA from soil microbes. This process involves the extraction of lipids, separation of phospholipids, and methanol esterification to isolate the PLFA from soil microbes. The resulting fatty acid methyl esters, dissolved in n-hexane after nitrogen blowdown, are analyzed using a MIDI Sherlock Microbial Identification System (MIDI Inc., Newark, DE, USA) coupled with a gas chromatograph (Agilent 7890B, Agilent, Palo Alto, CA, USA). The identification of individual PLFA components is achieved by matching their elution times with those of standards in the system’s library.

### Determination of carbon fractions and microbial necromass carbon contents

A 20 g portion of air-dried soil, passed through a 2 mm sieve, was weighed into a plastic bottle, and 100 mL of a 5 g L^−1^ solution of sodium hexametaphosphate was added. The bottle was placed on a reciprocating shaker (180 r/min) and oscillated for 18 h. The soil suspension is then passed through a 0.053 µm sieve, and the sieve is rinsed repeatedly with water. The coarse fraction remaining on the sieve is considered POC, while the fine fraction that passes through the sieve is considered MAOC.

Microbial necromass carbon (NC) is characterized using soil amino sugars. According to the acetyl derivative gas chromatography method of amino sugars described by Wang et al. (34), the content of amino sugars in soil samples is determined. After hydrolysis with hydrochloric acid, the samples are centrifuged, purified, and derivatized to obtain amino sugar derivatives. These derivatives are then analyzed using a gas chromatograph (Agilent 7890B GC, Agilent Technologies, Santa Clara, CA, USA).

### Statistical analysis

All data were analyzed using Microsoft Excel 2019. One-way analysis of variance (ANOVA) was performed using IBM SPSS Statistics, and plots were generated using R (version 4.2.3). The threshold for significance for one-way ANOVA was set at *P* < 0.05, and data were visualized using the ggplot2 package. R Studio (version 4.2.3) was utilized to perform principal component analysis (PCA), aiming to reveal variability patterns and assess the impact of each sampling site and variable on observed variances across different locations.

To unravel the processes behind SOC buildup under *P. parvifolia*, partial least squares path modeling (PLS-PM) was used. Using the NLME package for model fitting, we obtained *R*^2^ and *P* values. A range of potential models was produced by the modeling process, and the Akaike information criterion helped identify the optimal model using the dredge function in the R MuMIn package. The direction and extent of the linear connection between latent variables are shown by path coefficients. An adequate model fit is indicated by a goodness-of-fit ranging from 0.40 to 1.00 (33).

## RESULTS

### Relative concentrations of bacteria and fungi

**Fig 1:**
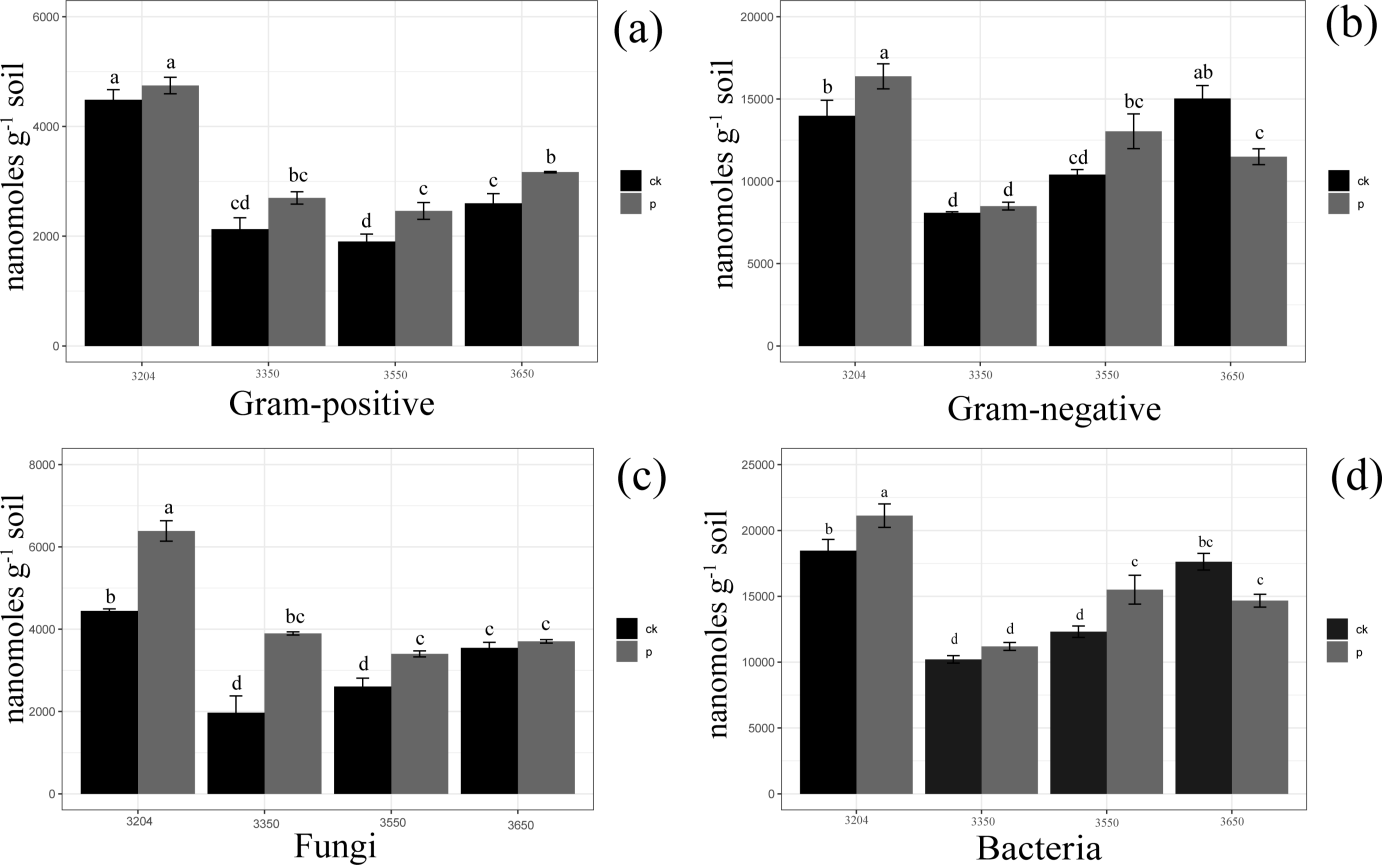
Relative contents of gram-positive bacteria (**a**), gram-negative bacteria (**b**), fungi (**c**), and bacteria (**d**) in the rhizosphere soil of *Potentilla parvifolia* (P) and bare soil (CK) at different altitudes. Data represent mean ± standard error (SE) of three independent replicates (*n* = 3). Different lowercase letters indicate significant differences among treatments at the same elevation according to one-way ANOVA followed by Tukey’s HSD test (*P* < 0.05).

The impact of *P. parvifolia* across altitudinal gradients on the rhizosphere soil gram-positive bacteria at different altitudes is shown in Fig. 1a and Tables 1 and 2. The experimental results indicate that the relative abundance of gram-positive bacteria increases at various altitudes compared to bare soil, with significant differences observed at altitudes of 3,350, 3,550, and 3,650 m (*P* < 0.05). The relative abundance of gram-positive bacteria in the rhizosphere soil of *P. parvifolia* is highest at an altitude of 3,204 m (4,747.33 nmol/g), while the relative abundance of gram-negative bacteria is lowest at an altitude of 3,550 m (2,461.34 nmol/g).

**TABLE 1:** Soil microbial content and carbon fractions content of *Potentilla parvifolia* rhizosphere soils at four elevations*^a^*^,^*^b^*

	P1	P2	P3	P4
pH	6.02 ± 0.07cd	6.08 ± 0.06c	6.15 ± 0.06b	6.25 ± 0.11a
TC (g/kg)	131.77 ± 3.04a	98.83 ± 2.45c	70.73 ± 0.24e	97.03 ± 3.51c
TN (g/kg)	9.63 ± 0.21a	6.13 ± 0.33d	3.9 ± 0.14f	6.4 ± 0.82cd
SOC (g/kg)	115.09 ± 1.74a	74.03 ± 1.10c	75.50 ± 1.19c	74.16 ± 1.82c
MBC (mg/kg)	1,048.33 ± 25.96a	529.15 ± 17.58d	477.93 ± 20.17de	851.16 ± 21.17b
POC (g/kg)	32.94 ± 1.65b	17.70 ± 3.00c	26.78 ± 1.34bc	27.48 ± 2.62bc
MAOC (g/kg)	57.63 ± 0.81a	30.33 ± 2.75cd	28.43 ± 2.57d	37.61 ± 1.41c
NC (mg/kg)	42,654.01 ± 1,604.82a	28,356.99 ± 443.97c	13,780.72 ± 591.01f	24,127.71 ± 755.79d

^*a*^ Values are mean ± SE (*n* = 3). Different letters within a row indicate significant differences among treatments (ANOVA, Tukey’s test, *P* < 0.05).

^*b*^ P1: rhizosphere soils of *Potentilla parvifolia* at 3,204 m; P2: rhizosphere soils of *Potentilla parvifolia* at 3,350 m; P3: rhizosphere soils of *Potentilla parvifolia* at 3,550 m; P4: rhizosphere soils of *Potentilla parvifolia* at 3,650 m.

**TABLE 2:** Soil microbial content and carbon fractions content of bare soils at four elevations^*a*^^,*b*^

	CK1	CK2	CK3	CK4
pH	6.12 ± 0.04bc	6.15 ± 0.06b	6.22 ± 0.07b	6.35 ± 0.08a
TC (g/kg)	112.83 ± 3.62b	61.27 ± 3.92f	82.93 ± 0.40d	86.57 ± 0.27d
TN (g/kg)	8.67 ± 0.09b	5.07 ± 0.31e	6.03 ± 0.12d	7.03 ± 0.12c
SOC (g/kg)	92.04 ± 2.84b	55.51 ± 1.67d	45.64 ± 1.18f	53.38 ± 1.05e
MBC (mg/kg)	834.94 ± 9.76b	451.39 ± 20.93e	441.44 ± 35.82e	729.78 ± 6.69c
POC (g/kg)	61.38 ± 3.36a	24.04 ± 2.38c	16.81 ± 2.41c	17.72 ± 2.95c
MAOC (g/kg)	49.44 ± 2.15b	33.58 ± 1.90cd	36.19 ± 2.09c	25.09 ± 2.86d
NC (mg/kg)	32,079.86 ± 278.58b	18,691.59 ± 525.34e	15,412.32 ± 527.49f	19,523.83 ± 538.53e

^*a*^ Values are mean ± SE (*n* = 3). Different letters within a row indicate significant differences among treatments (ANOVA, Tukey’s test, *P* < 0.05).

^*b*^ CK1: bare soils at 3,204 m; CK2: bare soils at 3,350 m; CK3: bare soils at 3,550 m; CK4: bare soils at 3,650 m.

The effects of altitudinal gradients of *P. parvifolia* on rhizosphere soil fungi at different altitudes are shown in Fig. 1c and Tables 1 and 2. The concentrations of fungi were higher in the rhizosphere soil than in the bare soil at various altitudes, with significant differences at 3,204, 3,350, and 3,550 m above sea level (*P* < 0.05). In the rhizosphere soil of *P. parvifolia*, fungal concentration peaked at 3,204 m with a measurement of 6,387.80 nmol/g and reached its minimum at 3,550 m with 3,399.75 nmol/g.

The effects of altitudinal gradients of *P. parvifolia* on rhizosphere soil bacteria at different altitudes are shown in Fig. 1d and Tables 1 and 2. The concentrations of the bacteria at medium and low altitudes (i.e., 3,204, 3,350, and 3,550 m) tended to be higher than those in bare soil, and the differences were significant at 3,204 and 3,550 m above sea level (*P* < 0.05). The bacterial content in the rhizosphere soil of *P. parvifolia* was highest at an altitude of 3,204 m (21,125.31 nmol/g) and lowest at 3,350 m (11,193.35 nmol/g).

### Soil organic carbon fractions

**Fig 2:**
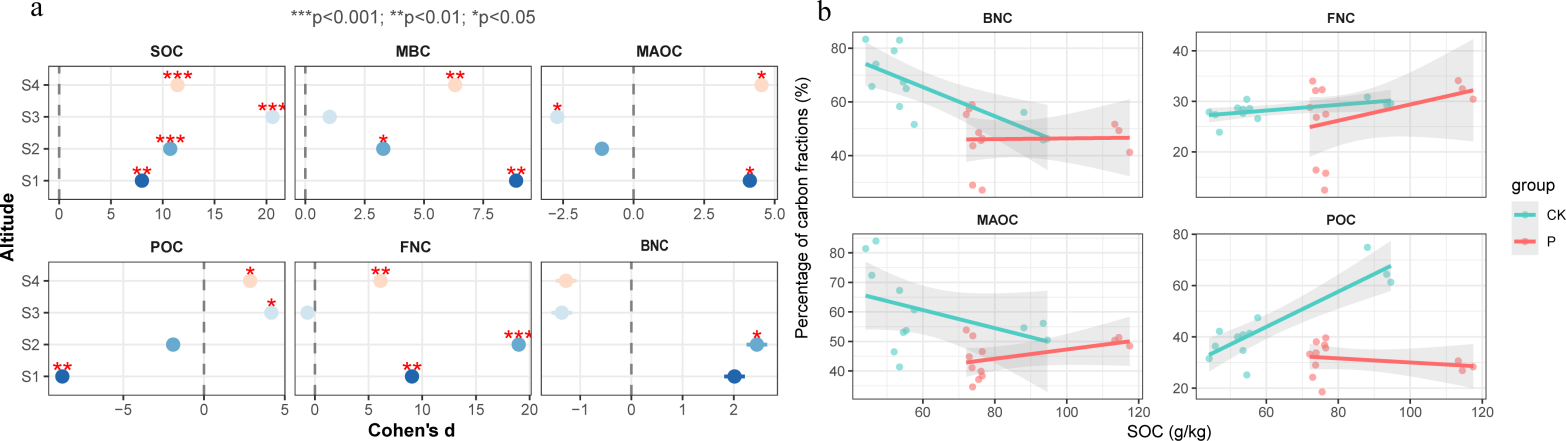
Effect of *P. parvifolia* on different carbon fractions at different altitudes (**a**) and the carbon fractions of P and CK vary with SOC (**b**) (*n* = 24).

Linear models of SOC and MAOC in the rhizosphere soil and bare soil without vegetation cover (CK) are shown in Fig. 2b and Tables 1 and 2. Compared to the estimates in the CK group (*R*² = 0.184, slope = −0.307), the contribution of MAOC to SOC was higher in the P group (*R*² = 0.199, slope = 0.158). As shown in Table 1, the MAOC content was the highest at an altitude of 3,204 m (57.63 g/kg) and the lowest (28.43 g/kg) at an altitude of 3,550 m.

Linear models of SOC and POC in the rhizosphere soil and bare soil without vegetation cover (CK) are shown in Fig. 2b and Tables 1 and 2. When compared with the estimates in the CK group (*R*² = 0.779, slope = 0.686), the contribution of POC to SOC in the P group was reduced (*R*² = 0.057, slope = −0.080). The POC content (32.94 g/kg) was highest at an altitude of 3,204 m and lowest (17.70 g/kg) at 3,350 m (Table 1).

As can be seen from Fig. 2a, *P. parvifolia* had a significant positive effect on SOC at all altitudes, and the effect value was the strongest at 3,550 m. *P. parvifolia* had a significant positive effect on MAOC at 3,204 and 3,650 m, and POC at 3,550 and 3,650 m. For fungal necromass carbon (FNC), there are significant positive effects at 3,204, 3,350, and 3,650 m, while for bacterial necromass carbon (BNC), there is a significant positive effect at 3,350 m.

### Contribution of microbial necromass carbon to soil organic Carbon

Linear models of SOC and microbial necromass carbon in the rhizosphere soil and bare soil without vegetation cover (CK) are shown in Fig. 2b and Tables 1 and 2. When compared to the estimates in the CK group (*R*² = 0.584, slope = −54.3), the contribution of bacterial necromass carbon to SOC increased in the P group (*R*² = 0.001, slope = 1.41). Compared to the estimates in the CK group (*R*² = 0.332, slope = 56.8), the contribution of fungal necromass carbon to SOC was higher in the P group (*R*² = 0.147, slope = 160). As shown in Table 1, the necromass carbon content in the rhizosphere soil of *P. parvifolia* was highest at an altitude of 3,204 m (42,654.01 mg/kg) and lowest at 3,550 m (13,780.72 mg/kg).

### Principal component analysis

**Fig 3:**
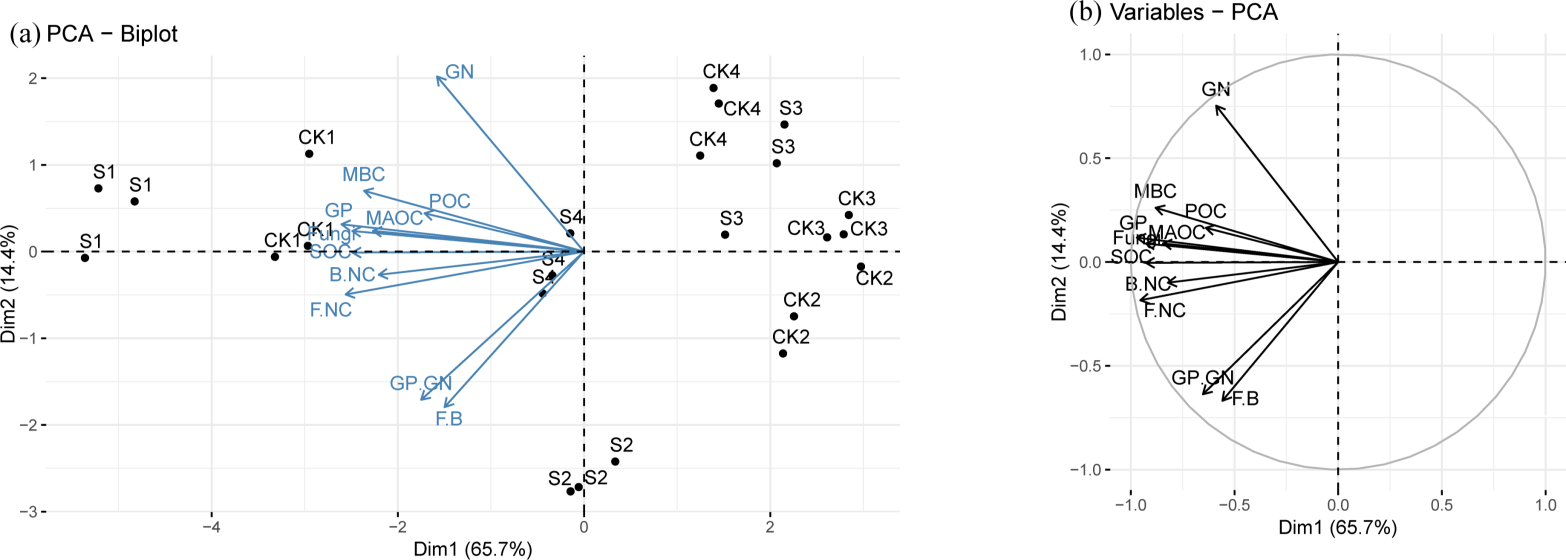
(**a**) The PCA score plot delineates the positions of sampling sites and the parameters assessed at each locale. (**b**) The carbon fraction’s influence on the initial two principal components is categorized into clusters.

The principal component analysis identified a clear relationship between the sampling site and the microbial community. As shown in Fig. 3a, we found significant differences among altitudes, and, as shown in Fig. 3a, along PC1, we observed that gram-positive bacteria and fungi were located near S1. These taxa were closer to samples from the low-altitude treatment and not related to samples from the high-altitude treatment. A distinct correlation was observed between the sampling site, microbial community, SOC composition, and microbial necromass carbon. As shown in Fig. 3a, along PC1, necromass carbon, microbial biomass carbon, SOC, and MAOC were closely related to gram-positive bacteria and fungi. As shown in Fig. 3b, these parameters had strong positive correlations with the low-altitude treatment but not with the high-altitude treatment.

### Partial least squares path modeling

**Fig 4:**
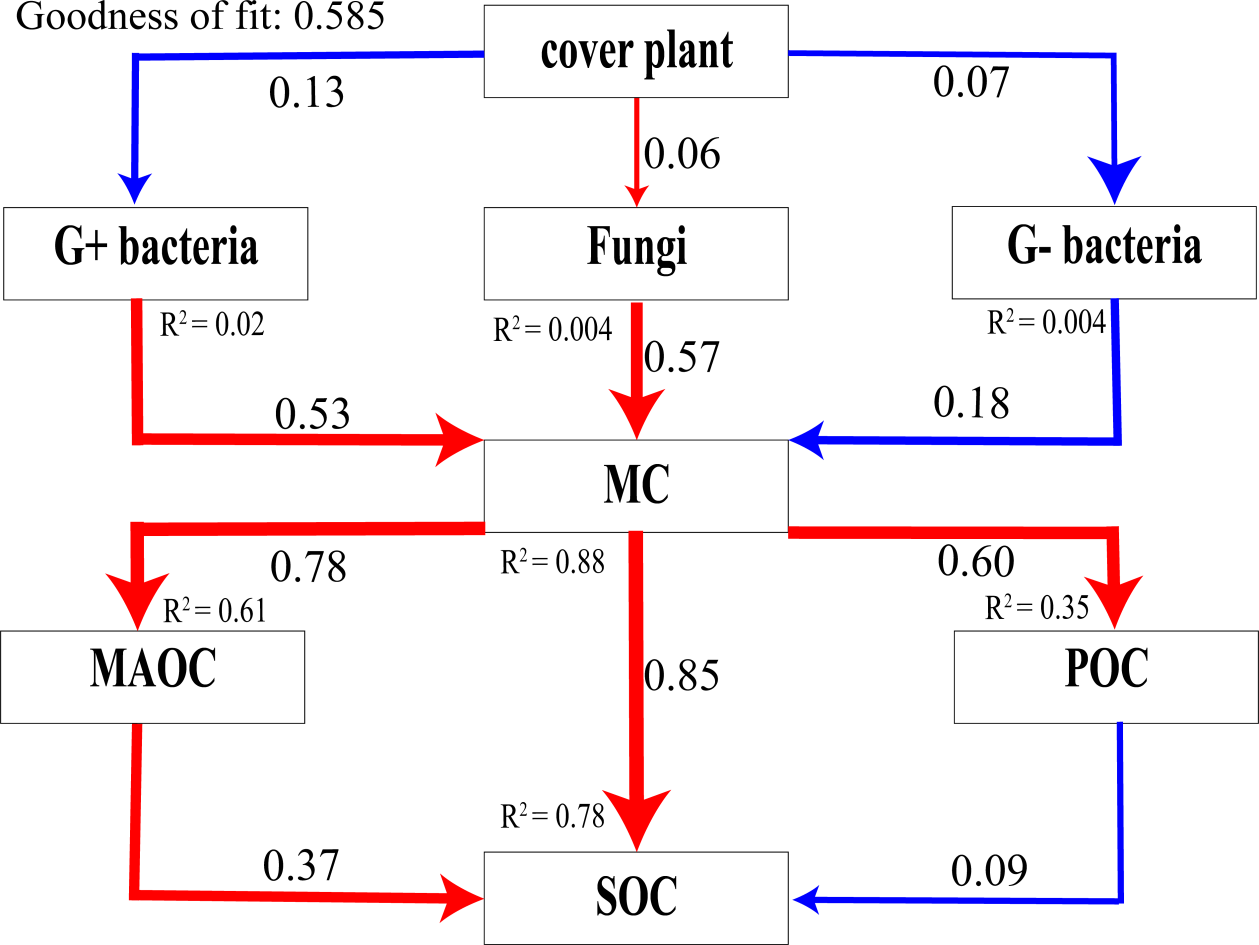
Positive correlations are represented by red lines, and negative correlations are represented by blue lines, with the thickness of the lines reflecting the strength of the effect. G+ bacteria refer to gram-positive bacteria. MC encompasses carbon of microbial origin, including both microbial biomass carbon and necromass carbon. G− bacteria refer to gram-negative bacteria.

The PLS-PM (Fig. 4) results indicated that gram-positive bacteria and fungi positively influenced the microbial-derived carbon pool. The microbial-derived carbon pool, in turn, positively affects MAOC, which positively impacts SOC. Although microbial-derived carbon also positively influences POC, the latter negatively affects SOC. However, the overall effect of microbial-derived carbon on SOC remained positive. This suggests that the presence of *P. parvifolia* enhances the capacity of soil to sequester organic carbon through its influence on carbon fractions.

### Mantel test

**Fig 5:**
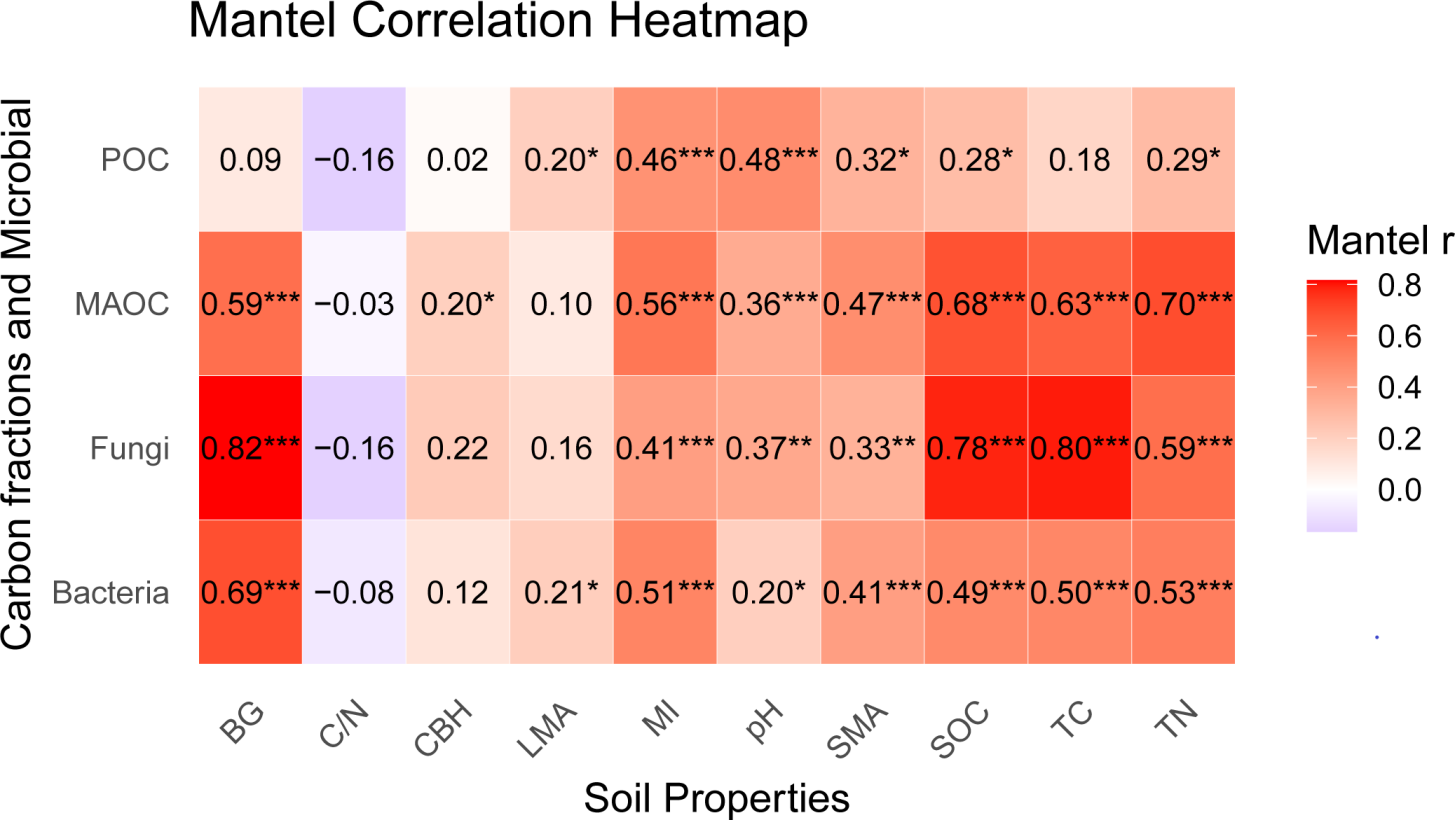
Mantel test heat map of fungi, bacteria, MAOC, POC, and physicochemical factors.

Mantel heatmap analysis (Fig. 5) revealed distinct environmental drivers between soil carbon fractions and microbial communities. POC showed significant positive correlations with microaggregate (MI), soil pH, mesoaggregates (SMA), organic carbon (SOC), and TN (*P* < 0.05). MAOC was positively correlated with β-glucosidase (BG), cellobiohydrolase, MI, pH, SMA, SOC, TN, and TC. Fungal abundance was primarily driven by BG, SOC, TC, and TN (*P* < 0.05), whereas bacterial abundance correlated significantly with BG, MI, TC, TN, and large macroaggregates macroaggregates (LMA). MAOC variation was predominantly regulated by BG, MI, SOC, TC, and TN, while POC exhibited weaker associations with physicochemical factors.

## DISCUSSION

### Soil microbial biomass changes at different elevations in the Qilian Mountains

In the rhizosphere of *P. parvifolia*, gram-positive bacteria, particularly Actinobacteria and Firmicutes, exhibited higher relative abundance compared to the surrounding bulk soil, suggesting a selective enrichment driven by rhizodeposits (1, 2). These taxa are generally regarded as K-strategists adapted to oligotrophic niches and root exudates, whereas gram-negative bacteria (e.g., Proteobacteria and Bacteroidetes) often act as r-strategists with rapid growth responses to labile carbon inputs (3). At higher elevations, environmental filtering further constrained bacterial community composition, consistent with previous findings that elevation strongly shapes bacterial assemblages (4).

Fungal relative abundance in *P. parvifolia* rhizosphere was consistently elevated across all altitudes, reaching peak significance at 3,204–3,350 m (*P* < 0.001). At these elevations, rhizodeposits likely stimulated symbiotic mycorrhizal networks, while shrub canopy interception reduced UV penetration, mitigating photoinhibition of fungal hyphae (5, 6). Above 3,550 m, melanin-rich fungal taxa, such as dark septate endophytes and melanized Ascomycota lineages, predominated—a known adaptation to high UV flux and oligotrophic conditions (7, 8). Their melanized cell walls and extracellular enzymes confer resilience and allow efficient decomposition of recalcitrant organic matter (7). In addition, AMF have been shown to persist in alpine ecosystems, indicating that *P. parvifolia* may sustain AMF associations even under extreme conditions (9).

### Effects of soil structural and fungal necromass carbon on carbon composition

The reversal of MAOC’s contribution to SOC in rhizosphere soils (slope shift from −0.307 in bare soil to 0.158 under *P. parvifolia*) correlates strongly with pH (*r* = 0.36) and MI dynamics (*r* = 0.56 with SOC). This suggests that root exudates modified rhizosphere microenvironments, enhancing mineral-organic binding through protonation or ligand exchange—a mechanism widely observed in alpine shrubs (35). Crucially, *P. parvifolia* mitigated the altitudinal MAOC decline seen in bare soils (slope = −0.307) by stabilizing MI, which physically protects MAOC from freeze-thaw-induced disaggregation (36). This contrasts with lowland systems, where macroaggregates dominate MAOC protection (37), underscoring the unique role of microaggregates in high-altitude carbon persistence. The observed MAOC peak at 3,204 m (57.63 g/kg) aligns with studies linking root exudate-mineral interactions to carbon stabilization in cold environments (38), while the MAOC minimum at 3,550 m (28.43 g/kg) coincides with the altitudinal peak of freeze-thaw frequency, where LMA disruption liberates POC for mineralization (39).

FNC dominated MAOC formation (PLS-PM path coefficient = 0.81), with its contribution to SOC tripling in rhizosphere soil (slope = 160 vs 56.8 in bare soil). This aligns with global studies emphasizing fungal residues’ chemical recalcitrance (40), but our data reveal altitudinal amplification: at 3,650 m, MAOC recovery coincided with peak FNC (Table 1), likely due to melanized fungal residues resisting UV and freeze-thaw degradation (7). Melanin, a key component of fungal necromass, forms stable organo-mineral complexes through aromatic interactions (41), a process critical for MAOC persistence in high-altitude soils (42). In contrast, BNC showed minimal SOC contributions except at 3,350 m (*P* < 0.05), reflecting bacteria’s transient role in alpine carbon cycling (43), where rapid turnover limits their stabilization potential (44).

The dual strategy of *P. parvifolia*—microaggregate reinforcement and fungal residue stabilization—counteracted carbon losses at 3,550 m, a critical “vulnerability zone” characterized by frequent freeze-thaw cycles. Enhanced physical protection of MAOC within microaggregates is evidenced by the strengthened correlation between MI and SOC, a mechanism analogous to cryoturbation-protected carbon in Arctic soils (45). Simultaneously, fungal necromass exhibited remarkable resistance to decomposition (FNC/SOC slope = 160), likely due to melanin’s recalcitrant properties (7). At 3,650 m, MAOC recovery under heightened UV stress (MAOC-TC correlation: *r* = 0.63) mirrors findings in Tibetan Plateau soils, where melanized fungi dominate carbon stabilization (46). These altitudinal patterns redefine alpine carbon sequestration as a process driven by fungal adaptations and microaggregate dynamics rather than macroaggregate-centric mechanisms typical of lowland ecosystems (37).

### Conclusion

*Potentilla parvifolia* increased soil microbial biomass, especially fungal biomass, at medium and low altitudes, in addition to significantly increasing SOC and TC content. Moreover, the contribution of MAOC to SOC significantly increased, with significant differences at medium and low altitudes, indicating that the refractory organic carbon content could be increased by increasing the MAOC in SOC contents and decreasing the POC in the SOC content, thereby influencing the carbon fractions. Microbial necromass carbon, especially fungal necromass carbon, had the greatest impact on soil MAOC, with significant differences seen between middle and low altitudes. These results suggest that *P. parvifolia* mainly alters the SOC fractions by increasing the fungal content in the microbial community, thereby achieving the goal of protecting the ecological environment of the Qilian Mountains.

## Data Availability

The data that support the findings of this study are available in the supplementary material of this article, and the code are openly available at https://github.com/LST-13/PLS.git.
